# Avian binocular vision: It’s not just about what birds can see, it’s also about what they can’t

**DOI:** 10.1371/journal.pone.0173235

**Published:** 2017-03-29

**Authors:** Luke P. Tyrrell, Esteban Fernández-Juricic

**Affiliations:** Purdue University, Department of Biological Sciences, West Lafayette, Indiana, United States of America; University of Sussex, UNITED KINGDOM

## Abstract

With the exception of primates, most vertebrates have laterally placed eyes. Binocular vision in vertebrates has been implicated in several functions, including depth perception, contrast discrimination, etc. However, the blind area in front of the head that is proximal to the binocular visual field is often neglected. This anterior blind area is important when discussing the evolution of binocular vision because its relative length is inversely correlated with the width of the binocular field. Therefore, species with wider binocular fields also have shorter anterior blind areas and objects along the mid-sagittal plane can be imaged at closer distances. Additionally, the anterior blind area is of functional significance for birds because the beak falls within this blind area. We tested for the first time some specific predictions about the functional role of the anterior blind area in birds controlling for phylogenetic effects. We used published data on visual field configuration in 40 species of birds and measured beak and skull parameters from museum specimens. We found that birds with proportionally longer beaks have longer anterior blind areas and thus narrower binocular fields. This result suggests that the anterior blind area and beak visibility do play a role in shaping binocular fields, and that binocular field width is not solely determined by the need for stereoscopic vision. In visually guided foragers, the ability to see the beak—and how much of the beak can be seen—varies predictably with foraging habits. For example, fish- and insect-eating specialists can see more of their own beak than birds eating immobile food can. But in non-visually guided foragers, there is no consistent relationship between the beak and anterior blind area. We discuss different strategies—wide binocular fields, large eye movements, and long beaks—that minimize the potential negative effects of the anterior blind area. Overall, we argue that there is more to avian binocularity than meets the eye.

## Introduction

Despite most vertebrates having their eyes positioned laterally rather than frontally, the discussion about binocular vision in vertebrates has traditionally revolved around the extraction of relative depth information from stereopsis [1–3]. Even human stereopsis is only practical over a short range from the observer [4]. At long distances, depth information is acquired by different mechanisms that may be performed monocularly, such as interposition and motion parallax (reviewed in [3]). Yet other functions of binocularity, such as increased light sensitivity and contrast discrimination, have been less studied (reviewed in [3]). All birds have binocular vision, but evidence for binocular stereopsis only exists in barn owls (*Tyto alba*) and a few other birds of prey [5,6]. Multiple factors, including disconjugate eye movements [7,8], seem to suggest that stereopsis is not present in most bird species despite the presence of a binocular overlap [9,10]. The fact that depth information is available in the absence of stereopsis [11], coupled with the existence of binocularity in species that likely lack stereopsis [12], certainly leaves room for the idea that binocular vision is about much more than stereopsis and depth perception.

**Fig 1:**
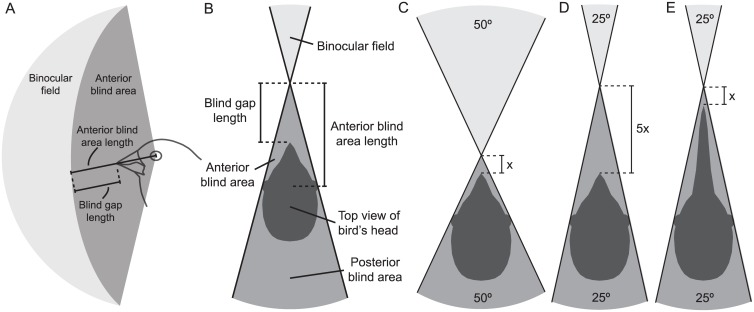
Anterior blind area of the avian visual field. (A) Side view of a vertical section through the avian binocular field and anterior blind area, also show the anterior blind area length and blind gap length. (B) Top view of a horizontal section through the avian visual field. The lines protruding from the eyes mark the edge of each eye’s visual field. The anterior blind area is the shaded space that extends from the eyes to the beginning of the binocular visual field. The anterior blind area in this schematic representation encompasses the beak. The blind gap length is a subsection of the anterior blind area that extends from the beak tip to the beginning of the binocular visual field. (C-D) As the binocular field becomes narrower from C to D, the length of the anterior blind area becomes longer but the posterior blind area becomes smaller. If the head width is held constant, then a two-fold increase in the binocular field width results in a five-fold decrease in blind gap length. (E) But increasing beak length can shorten the blind gap.

Martin [10] proposed that one primary function of avian binocular vision is the visual control of the beak in many species or the feet in the case of raptors [e.g., 13,14]. Species that peck or lunge at prey, that build intricate nests, or that feed atricial young would benefit by estimating time to contact using symmetrically expanding optic flow information from their binocular fields [10]. Vision along the mid-sagittal plane is binocular vision because birds possess bilateral symmetry. Therefore, the beak—which falls along the mid-sagittal plane—is generally viewed with binocular vision. Martin [10] argued that even species with visual control of the beak have a blind area immediately anterior to the head, just before the binocular field begins (Fig 1). This *anterior blind area* occurs because there is a gap between the eyes that lie on either side of the mid-sagittal plane (Fig 1A and 1B). There are three relevant components of this anterior blind area in a two-dimensional plane. First, there is the *anterior blind area* itself, which is defined as the area between the eyes to the start of the binocular field that does not have any visual input (Fig 1A and 1B). Second, there is the *anterior blind area length*, which is the linear distance from the point on the mid-sagittal plane between the nodal points of each eye to the start of binocular field (Fig 1A and 1B). Third, there is the *blind gap length*, which is defined as the linear distance from the beak tip to the start of the binocular field (Fig 1A and 1B).

The anterior blind area has the potential to be a driver in the evolution of binocular vision because there is a geometric relationship between binocular field width and anterior blind area length. As binocular field width increases, not only does the area covered by both eyes become larger, but the anterior blind area also becomes shorter and consequently the binocular field is brought closer to the beak (Fig 1C and 1D). Yet the degree of interspecific variation and function of this anterior blind area has often been overlooked when discussing avian binocular vision.

In this study, we argue that the relative length of this anterior blind area is of functional relevance for birds because the beak falls within this anterior blind area and is fixed in one position. Without forelimbs designed for grasping, the beak is the bird’s primary organ for physically interacting with the environment (e.g., manipulating and grabbing food, feeding nestlings, preening, interacting with mates, defending territories, mobbing predators). It is this reliance on the beak that makes the avian case special in regards to the anterior blind area. Functionally, an anterior blind area creates a space around the beak where there is a lack of information that may be important for visually controlling the beak as it approaches a target. The longer the blind gap length, the longer the distance that birds would need to estimate (rather than track visually) and the longer the time of uncertainty before the beak reaches the target (e.g., food, substrate, mouth of nestlings, conspecific).

We propose that in bird species requiring visual control of the beak, the length of the blind gap would be relatively small to minimize the spatial and temporal uncertainty while interacting with the physical world. The benefit of reducing the blind gap length would be particularly pronounced in species that keep their eyes open and continuously accommodate their lenses to visually track their targets during pecking (e.g., chickens [15,16]). Other species use ballistic pecking where the eyes are closed and the final phase of the peck consists of a stereotyped motion (e.g., pigeons [17,18]). Even these ballistic species would benefit from reducing their blind gap length because pecking accuracy is diminished when the ability to estimate time/distance to contact is experimentally reduced [19], but only until it is equal to the length of their non-visual ballistic phase. In other bird species, their blind gaps can be so short that they actually gain visibility of the beak tip with their binocular fields, which is expected to enhance their ability to manipulate objects (e.g., food items or even tools to retrieve difficult-to-access items [20,21]). Some birds are further specialized for frontal vision by having a ‘ramp retina’ that allows the bird to simultaneously focus on close, frontally located objects (i.e., objects close to the beak) and distant, laterally located objects [22].

On an evolutionarily scale, the anterior blind area would be shorter when orbits are rotated forward in the skull, which would increase binocular field width [23,24]. Additionally, species with longer beaks could also have shorter blind gaps (Fig 1E). On an individual scale, a bird could temporarily reduce the length of the anterior blind area by making convergent eye movements bringing the binocular field closer to the beak. Alternatively, an individual or species could increase the velocity of its peck or lunge to reduce the length of its blind gap in time rather than distance. On the other hand, some species with long anterior blind areas may not compensate at all because they do not require visual control of the beak (e.g., tactile foragers, filter feeders). We developed some predictions that are derived from these hypotheses.

We corroborated with empirical data the geometric relationship by which an increase in binocular field width is associated with a decrease in the anterior blind area length. As a result, we predicted that the binocular field would be brought closer to the beak, leading to a negative relationship between binocular field width and blind gap length. Because longer beaks extend further out from the head and are easier to see, having a long beak would also relax the pressure to bring the eyes forward to reduce anterior blind area length. Therefore, we predicted that species with longer beaks would also have longer anterior blind areas and thus narrower binocular fields.

Given the large interspecific differences in foraging techniques and diets, we also made predictions for different relationships between the length of the anterior blind area and beak morphology depending on foraging requirements, sorting species into four groups (pecking foragers, non-raptorial predators, diurnal raptors, and tactile/filter foragers). We predicted that pecking foragers would be able to tolerate some amount of blind area in front of the beak tip because their food/prey items do not move (or move very slowly). Instead, they can use the symmetrically expanding optic flow-field in the direction of travel to anticipate time to contact [10], and seeing the target in the final moments before contact may not be necessary. For non-raptorial predators (i.e., insectivorous and piscivorous that are non-raptorial, meaning they capture prey with their beak rather than “raptorial” appendages like talons), a large anterior blind area in front of the beak would be detrimental to prey capture because quick, erratic flight by their prey could take it out of danger in the time it takes the bird to close the remaining blind gap between the margin of the visual field and the beak. Therefore, we expect non-raptorial predators to have smaller blind gap lengths than pecking foragers. Non-raptorial predators with short beaks would need a shorter anterior blind area (i.e., wider binocular field) to bring vision in towards the beak tip, whereas non-raptorial predators with longer beaks could still see the beak tip with a longer anterior blind area (i.e., narrower binocular field).

We predicted that species requiring more visual control of the beak (pecking foragers, non-raptorial predators) would use eye movements more to temporally manipulate the size of their anterior blind area compared to species that do not require considerable visual control of the beak. Consequently, species with more visual control of the beak would be able to see their beak tips (i.e., enhancing visual manipulation) more often when they converge their eyes forward rather than when their eyes are in the resting position.

Diurnal raptors, or birds of prey, share many characteristics with non-raptorial predators in terms of the visual information needed to detect and pursue prey [25,26]; however, we predicted that diurnal raptors can afford longer blind gaps in front of the beak because they grasp their prey with their talons, which are further away from the eyes than the beak tip. Finally, tactile foragers and filter feeders have little or no need for visual control of foraging [9,10]; thus, we predicted that these species would have a large variation in blind gap length, their anterior blind area size would be unrelated to beak length, and their binocular fields would be the narrowest of all groups considered.

In this study, we tested these predictions for the first time using multiple species of birds and controlling for the effects of phylogenetic relatedness. We used published data on visual field configuration, and gathered beak and skull measurements from museum specimens.

## Methods

We compiled data from the literature on the binocular field width from 40 species of diurnal birds (see list in S1 Table), measured different aspects of the beak and skull morphology from museum specimens, and calculated dimensions of the anterior blind area to test the relationships between different traits, controlling for the effects of phylogenetic relatedness. All anterior blind area measurements were made in a two-dimensional plane that intersects the beak tip and the center of both eyes.

We gathered data from the literature on binocular field width with the eyes in a converged position and when the eyes were in a resting position. All of the literature sources used the ophthalmoscopic reflex technique to measure the retinal visual field. In this technique, an observer views a single eye through an ophthalmoscope and moves around the perimeter arm of a visual field apparatus (see [27]) until the retinal reflex disappears from the ophthalmoscope viewfinder. This position of disappearance corresponds to the extent of the retinal visual field. The process is repeated for the second eye, and the combination of the two retinal margins yields the binocular field width—or blind area width—in degrees. All binocular field widths used in this study were taken along the plane connecting the eyes to the beak tip because this is the most relevant plane for visual control of the beak. Unless otherwise stated, calculations used the binocular field width with the eyes converged because this is the state adopted by birds when foraging and using visual control of the beak [8,16,28–30] and is reported in the literature more frequently than the resting state. If the beak blocked the view of the eye during visual field measurements, then we used extrapolated binocular field values that reasonably assumed that the edge of the retina followed a gradually curving path [31,32].

**Fig 2:**
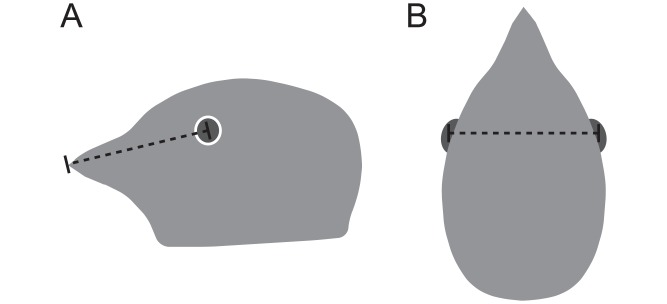
Skull measurements. (A) Side view of the head with a dotted line representing the distance between the focal point of the eye and the beak tip. (B) Top view of the head representing internodal distance.

Using museum specimens, we measured distance from the eye to the beak tip as the linear distance along the mid-sagittal plane connecting the center of the orbit to the beak tip (Fig 2A). Internodal distance is the linear distance between the focal points of each eye (Fig 2B). We obtained a proxy for this distance by measuring the skull width just posterior to the *orbitale superius* that horizontally aligned with the *optic foramen*. We also measured beak length (the posterior end of the nares to the beak tip) and skull width (widest point posterior to the orbits). All represented skull measurements are the average of multiple individuals with equal numbers of males and females within each species (sample sizes in S1 Table). Nearly all skull measurements were made using the skeletal collections at the Field Museum of Natural History in Chicago, IL, USA. The exceptions were *Malacorhynchos membranaceus*, *Puffinus puffinus*, and *Corvus monoduloides*, for which we measured skulls that were inside prepared skins from the same museum. Museum information and specimen numbers can be found in S1 Table.

We calculated anterior blind area length and blind gap length in all 40 bird species for which both visual field data were available in the literature and skull measurements could be obtained at the Field Museum of Natural History in Chicago, IL, USA. Anterior blind area length was calculated as:

$$anterior\;blind\;area\;length=\;\frac{\frac{1}{2}\;internodal\;distance}{\text{tan}\left(\frac{1}{2}\;binocular\;field\;width\right)}$$

Blind gap length was calculated as:

$$blind\;gap\;length=\;\frac{\frac{1}{2}\;internodal\;distance}{\text{tan}\left(\frac{1}{2}\;binocular\;field\;width\right)}-distance\;from\;eye\;to\;beak\;tip$$

Positive values for blind gap length indicate the presence of a blind gap in front of the beak, negative values for blind gap length indicate that the beak can been seen with the binocular field, and a value of zero would indicate that the binocular field starts exactly at the tip of the beak.

### Statistical analyses

We used phylogenetic generalized least squares (PGLS) regressions to test for correlations while controlling for phylogenetic effects in R (version 3.3.0) using the pgls() function. The phylogenies used in the analyses were obtained from http://birdtree.org using the Ericson All Species backbone [33]. We generated 2,000 trees from http://birdtree.org, from which we generated a single consensus tree to use in the PGLS regressions by using the 50% majority-rule in the SumTrees program within the Python (version 2.7) package DendroPy (version 4.0.0) [34,35].

PGLS is designed to work best with continuous explanatory factors. Therefore, comparisons between different foraging groups were carried out using dummy variables of 0 and 1 rather than discrete ‘pecking’ and ‘raptor’, for example. The only statistics not using PGLS were tests of whether tactile foragers had significantly more variation in blind gaps than other groups. Those tests were done using the var.test() function in R. In all PGLS results, t-values given by R were converted to F-statistics using t^2^ = F, which is appropriate because the numerator degrees of freedom is 1. The variables anterior blind area length and blind gap length were standardized for differences in head size by dividing each variable by skull width. Certain variables were log base-10 transformed to meet the normality assumptions of the model. If variables included a value between 0 and 1, then the entire variable was log transformed as log10(variable + 1 –min(variable)). Because binocular width and standardized anterior blind area length are geometrically associated to one another, they were never included as co-explanatory variables in the same regressions to avoid violating model assumptions. Standardized anterior blind area length and standardized blind gap length could be included together without violating assumptions (R^2^ < 0.6).

## Results

**Fig 3:**
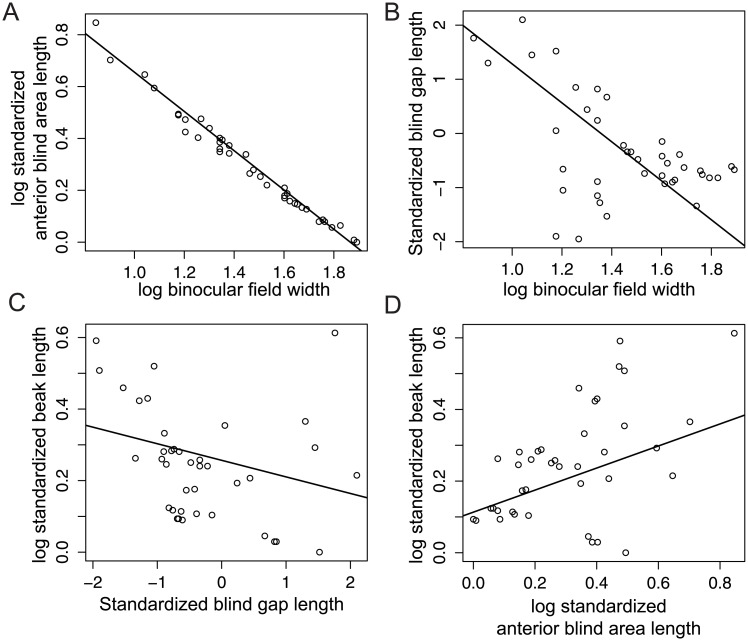
The relationships between the binocular vision, blind areas, and beak size. (A) Species with wider converged binocular fields (in degrees) have shorter anterior blind areas and (B) shorter blind gaps. (C) Species with longer beaks have shorter blind gaps, even though (D) they have longer anterior blind areas. All four correlations depicted are significant (Table 1). All length measurements were standardized for differences in head size by dividing each variable by skull width.

Considering all bird species studied and controlling for beak length (Table 1), we found that as binocular field width increases, the length of the anterior blind area became shorter (Table 1; Fig 3A). This led to a reduction in the blind gap length with an increase in binocular field width (Table 1; Fig 3B). Additionally, species with longer beaks had significantly shorter blind gaps (Table 1; Fig 3C) despite an increase in total length of the anterior blind area (Table 1; Fig 3D) and a narrower binocular field width (Table 1).

**Table 1:** PGLS regression results.

Response variable	Explanatory variables	d.f.	F	P	λ
log(binocular width) ~	log(standardized ABAL)	1,36	234.90	< 0.001	0.74
	+ standardized BGL	1,36	5.47	0.025	
	+ log(standardized beak length)	1,36	6.18	0.018	
log(standardized beak length) ~	standardized BGL	1,37	125.68	< 0.001	0.97
	+ log(standardized ABAL)	1,37	115.43	< 0.001	
log(standardized beak length) ~	standardized BGL	1,37	71.59	< 0.001	0.95
	+ log(binocular width)	1,37	62.54	< 0.001	
standardized BGL / standardized beak length ~	foraging group(pecking, predator)	1,29	5.92	0.021	0.98
log(standardized beak length) of foraging group(predator) ~	log(standardized ABAL) of foraging group(predator)	1,8	20.61	0.002	0.00
standardized BGL—standardized resting BGL ~	foraging group(pecking:predator, raptor:tactile)	1,38	4.31	0.045	0.65
standardized BGL ~	foraging group(predator, raptor)	1,12	35.66	< 0.001	0.00
log(standardized beak length) of foraging group(tactile) ~	log(standardized ABAL) of foraging group(tactile)	1,3	0.45	0.549	0.00
log(binocular width) ~	foraging group(tactile, all others)	1,38	7.14	0.011	0.95

The λ coefficient is a measure of the phylogenetic signal in the regression. λ-values close to 0 indicate no phylogenetic effects, whereas values close to 1 suggest a Brownian motion model of evolution. Anterior blind area length and blind gap length are abbreviated ABAL and BGL, respectively. Foraging group(predator) refers to non-raptorial predators. All length measurements were standardized for differences in head size by dividing each variable by skull width.

We predicted that pecking foragers would not be able to see their own beak tip because they would not necessarily need to see their beak to visual control it. We predicted that non-raptorial predators would be able to see their beak tip because they have more active prey that are difficult to target. Putting these two predictions together, we also expected non-raptorial predators to be able to see more of their own beaks than pecking foragers. We actually found that both pecking foragers and non-raptorial predators could see their beak tips on average because they both have negative blind gap lengths (standardized blind gap length = -0.32 ± 0.17 and -0.99 ± 0.60, respectively). However, the degree to which the beak was visible to these two groups varied. Non-raptorial predators were able to see the distal 74% (±7%) of their beaks, leaving only the most basal regions near the nares in the anterior blind area. This was significantly more than in pecking species, whose beak visibility was limited to the tip of the beak (38% ± 16%; Table 1).

Non-raptorial predators with increasingly shorter beaks had shorter anterior blind area lengths—and consequently wider binocular fields—relative to similar species with increasingly longer beaks (coefficient = 0.79; Table 1). Groups of birds that regularly use visual control of the beak (non-raptorial predators and pecking foragers) were able to manipulate the size of their blind gap through eye movements significantly more than other groups of birds (diurnal raptors and tactile/filter foragers), which may not require visual control of the beak at anytime (Table 1).

**Fig 4:**
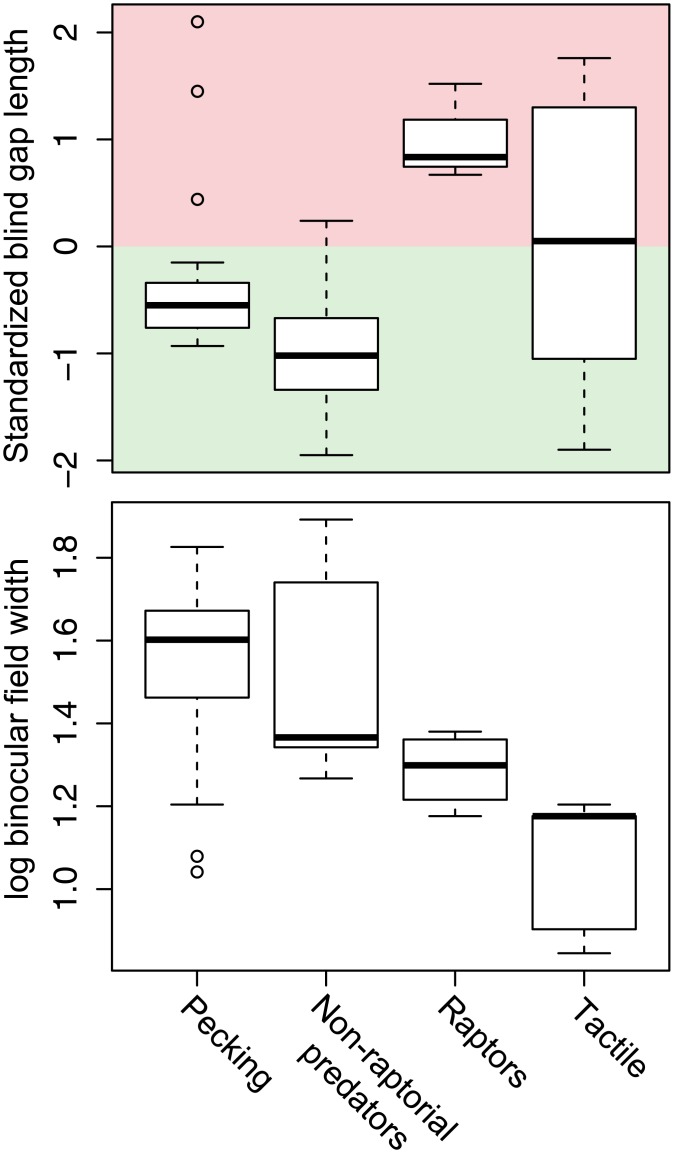
Foraging strategy predicts blind gap length and binocular field width. Box and whisker plots showing blind gap length (standardized for skull width) and converged binocular field width in four groups of birds with different foraging strategies. Standardized blind gap lengths that are positive (red) indicate that beak is not visible, whereas negative values (green) indicate that the beak is visible.

Diurnal raptors had large anterior blind areas that prevented them from seeing their beak (standardized blind gap length = 0.97 ± 0.19; Fig 4), and significantly longer blind gap lengths than non-raptorial predators (Table 1; Fig 4).

Tactile foragers and filter feeders had significantly greater variation in blind gap length than other groups of birds (ratio of variances = 0.25, P = 0.031, pecking species; ratio of variances = 0.15, P = 0.019, non-raptorial predators; ratio of variances = 0.06, P = 0.044, raptors; Fig 4). Additionally, we found no significant relationship between their anterior blind area length and beak length in tactile foragers and filter feeders (Table 1). Finally, tactile foragers had the narrowest binocular fields of all groups of species considered (Table 1; Fig 4).

## Discussion

Our results show that the anterior blind area and the blind gap vary substantially between species. Morphological differences in beak size, binocular field size, and foraging requirements appear to influence the length of the anterior blind area in different species. The length of the anterior blind area becomes shorter with an increase in binocular field width (Fig 3A) because the area covered by both eyes become wider and the origin of the binocular field is closer to the beak (Fig 1C and 1D). There are different ways the length of the anterior blind area can be reduced. Having the eyes more frontally placed within the skull increases the width of the binocular field [23,24] and thus decreases the length of the anterior blind area. Alternatively, the large convergent eye movements (20°-40°) that many bird species are capable of [8,36,37] can temporarily reduce the length of the anterior blind area [38].

Rather than an evolutionary repositioning of the orbits or an individual moving its eyes, we found that some species obtain shorter blind gaps by virtue of having long beaks that extend out toward the binocular field (Fig 1D and 1E). Remarkably, species with longer beaks have these shorter blind gaps (Fig 3C) despite an increase in the total length of the anterior blind area (Fig 3D) and a narrower binocular field. In addition to being easier to see, long beaks can also be used to probe deep into substrates for food that would be otherwise unreachable, a common tactic among shorebirds and some grassland birds. However, longer beaks also have lower overall crushing force, making them potentially costly [39].

There is a geometric trade-off between the width of the binocular field (and consequently the length of the anterior blind area) and the blind area that occurs behind the head. As the binocular field widens and the length of the anterior blind area shortens in front of the head, the blind area behind the head becomes wider, reducing overall visual coverage (Fig 1C and 1D). Reduced visual coverage may have negative fitness consequences, including lower probabilities of predator detection and gathering social information from group mates [40–42]. Therefore, the size of the blind area behind the head is expected to be minimized unless a greater benefit could be gained by increasing binocular field width or by reducing anterior blind area length. The position of the eyes in the skull is under multiple constraints, including beak visibility, blind areas, and aerodynamics. Eye movements could provide enough flexibility to get the best of both worlds. During flight and antipredator vigilance, a bird may have its eyes in a lateral resting position, which would provide a relatively large cyclopean field of view (i.e., total area of visual coverage around the head), but relatively limited visual control of the beak. However, at the moment before pecking or chick provisioning, the eyes could be swung forward to reduce the length of the anterior blind area and gain better visual control of the beak [8,29,30].

Indeed, we find that eye movements play a crucial role for pecking foragers and non-raptorial predators, which both rely on visual control of the beak to some extent. On average, neither group can see their beaks when the eyes are at rest, but both groups gain beak visibility when the eyes are swung forward. The total proportion of their daily time budget that requires fine beak control is relatively low. Therefore, both groups make use of large amplitude eye movements to visualize the beak while foraging but maintain a large cyclopean field of view while vigilant. For example, pigeons can only see their beak when their eyes are converged, and behavioral experiments have shown that pigeons swing both eyes forward to a converged position when pecking at seeds [29]. A recent comparative study on Emberizid sparrows found that species with wide binocular fields (hence, already short anterior blind areas) tend not to have a large degree of eye movement; however, species with narrow binocular fields have large convergent eye movements that increase binocular width, and reduce the relative size of the anterior blind area and gain better control over the beak [38]. In fact, these pecking birds and non-raptorial predators are able to manipulate the size of their anterior blind area through eye movements significantly more than other groups of birds that may not require visual control of the beak at anytime (Table 1).

If fine manipulation or identification of captured food items is required, then visibility of not only the beak tip but also the beak edges where manipulation occurs may be important. Starlings [22] and meadowlarks [30], for example, use an open-beak probe foraging strategy where the long beak is inserted into the ground and opened to expose food items beneath the surface. The eyes are then swung forward allowing starlings and meadowlarks to see nearly the entire beak (S1 Table), or perhaps more importantly, to visually inspect the exposed area between the mandibles [22,30]. And starlings—at least—also have a ‘ramp retina’ to better accommodate on items between the mandibles. Bird species that use tools, such as New Caledonian crows (*Corvus moneduloides*) and American crows (*Corvus brachyrhynchos*), have particularly wide binocular fields and can see their beak tips [20,21]; hence, their anterior blind areas are small. In the case of the New Caledonian crow, seeing along the shaft of the tool appears to be the key (rather than binocular vision) for using tools to extract food from long, narrow holes in wood [21,43]. When the tool is being used inside a narrow hole, it is held at an angle so that there is an ipsilateral eye and a contralateral eye, but only the contralateral eye can see the tool tip [43]. Without a wide binocular field that can see the beak tip, the crow would not be able to see the tool tip at all. Seeing their beaks also allows crows to control and manipulate the base of the tool [21].

Non-raptorial predators, such as insectivores and piscivores, have the need to visually guide mobile prey into their beaks, consequently the anterior blind area length of species with shorter beaks is shorter, hence increasing binocular overlap, than species with longer beaks. Even more interesting is that non-raptorial predators can see a larger proportion of their beaks than pecking foragers, which underscores the relevance of beak visibility in prey capture, identification, and manipulation tasks. Cormorants, for example, use visual control of the beak to expertly manipulate fish so that their fins are pointing backwards to make swallowing easier [13].

As predicted, we found that diurnal raptors had a longer blind gap between the beak and the start of the visual field than non-raptorial predators (Fig 4). Like other predators, raptors must detect live prey at a distance before the prey detects the raptor [42] and then visually track the prey during pursuit to judge relative speed, distance and trajectory before finally capturing the prey [25,26]. But because raptors grasp prey with their talons instead of their beak, they can still be successful with a longer blind gap. The extended talons are much further away from the eyes than the tip of the beak. So despite diurnal raptors’ narrow binocular fields, they are still able to see their extended talons in the binocular visual field [14,44,45].

We found that tactile/filter foragers have the greatest variation blind gap length (Fig 4) and have no consistent or meaningful relationship between the length of the anterior blind area and the beak. They do, however, have the narrowest binocular fields of any group (Fig 4). These species make use of mechanoreceptors in the beak, and therefore do not require fine control over beak placement or are able to do so using information from touch rather than vision. Woodcocks (*Scolopax* spp.), for example, use touch receptors concentrated in the tip of the beak to detect and capture invertebrates within soft substrates [46,47], and they have no binocular vision along the beak plane at all [47]. The reduced importance of binocular vision in these species introduces the opportunity to increase the probability of predator detection by reducing the size of the blind area behind the head, as we found in this study. In fact, Guillemain et al. [48] found that filter feeding Northern Shovelers (*Anas clypeata*) have more comprehensive cyclopean visual coverage around the head than their visually foraging congeners, Eurasian Wigeon (*Anas penelope*). The extra visual coverage pays off behaviorally, allowing the shovelers to spend less time vigilant and more time foraging [48]. There are some exceptions where filter feeders may need visual control over the beak. For example, flamingos have altricial young that are fed by adults. Adult flamingos are able to see their beak tips because they require fine control over beak placement as they drip food into chicks’ open mouths [49].

The literature to date has tried to understand avian binocular vision by comparing the size of the binocular field across different species, which has led to some fruitful hypotheses [9,10,38]. However, this emphasis has neglected to consider the potential role of the anterior blind area in reducing the amount of visual information in front of the beak. Our study reveals that species with wider binocular fields also have shorter anterior blind areas and shorter blind gaps. Beak length also plays an important role in shaping binocular fields, as species with relatively long beaks have narrower binocular fields yet still maintain shorter blind gaps than species with short beaks. Furthermore, foraging mode has an effect on the relationship between the beak and visual field. Visual guided foragers have consistent relationships between their visual field configurations and their beaks depending on the specific visual requirements of their foraging tasks. Non-visually guided foragers, however, are highly variable and have no consistent relationship between their beak and visual field configuration. We propose that the anterior blind area is a complementary (rather than mutually exclusive) functional explanation to variation in avian binocular vision.

## Supporting information

S1 Table Data table for the 40 species used in analyses. Units for binocular field widths are in degrees. Unless specifically noted, values were calculated using converged binocular field widths. Units for the distance from focal point to peak tip, internodal distance, and skull width are in mm. Standardized parameters were divided by skull width to control for body size. Negative values of standardized blind gap indicate the ability to see the beak tip and positive values indicate the inability to see the beak tip. If no resting binocular state was reported in the literature, we calculated it as the extrapolated converged binocular–(0.33 * (extrapolated converged binocular—diverged binocular)) which is the proportion for starlings in Martin 1986.
(DOCX)
