# Supplementary material for: Avian binocular vision: It’s not just about what birds can see, it’s also about what they can’t
Source: PLoS One. 2017 Mar 29;12(3):e0173235. doi: 10.1371/journal.pone.0173235 (PMC5371358; doi:10.1371/journal.pone.0173235)
Supplement: S1 Table — Units for binocular field widths are in degrees. Unless specifically noted, values were calculated using converged binocular field widths. Units for the distance from focal point to peak tip, internodal distance, and skull width are in mm. Standardized parameters were divided by skull width to control for body size. Negative values of standardized blind gap indicate the ability to see the beak tip and positive values indicate the inability to see the beak tip. If no resting binocular state was reported in the literature, we calculated it as the extrapolated converged binocular–(0.33 * (extrapolated converged binocular—diverged binocular)) which is the proportion for starlings in Martin 1986. (DOCX) [file pone.0173235.s001.docx]

S1 Appendix for:

Avian binocular vision: it’s not just about what birds can see, it’s also about what they can’t

*PLoS One*

Luke P. Tyrrell^1,2^ & Esteban Fernández-Juricic^1^

^1^ Purdue University, Department of Biological Sciences, 915 W. State St. West Lafayette, IN 47904

^2^ Corresponding author: ltyrrell@purdue.edu

| ***Common Name*** | ***Latin Name*** | ***Foraging group*** | ***n*** | ***Binocular width*** | ***Standardized ABAL*** | ***Standardized blind gap*** | ***Standardized beak length*** | ***Resting binocular width*** | ***Standardized blind gap with eyes at rest*** | ***Distance from focal point to beak tip*** | ***Internodal distance*** | ***Skull width*** |
| --- | --- | --- | --- | --- | --- | --- | --- | --- | --- | --- | --- | --- |
| Coopers Hawk | *Accipiter cooperii* | raptor | 4 | 24^u^ | 1.70 | 0.67 | 0.51 | 24^u^ | 0.67 | 33.87 | 23.88 | 33.04 |
| Northern Shoveler | *Anas clypeata* | tactile | 4 | 15^d^ | 2.43 | -1.90 | 2.62 | 15^d^ | -1.90 | 92.14 | 13.61 | 21.25 |
| Eurasian Wigeon | *Anas penelope* | pecking | 2 | 16^d^ | 2.00 | -0.66 | 1.31 | 16^d^ | -0.66 | 64.29 | 13.59 | 24.17 |
| Mallard | *Anas platyrhynchos* | tactile | 4 | 8^h^ | 4.38 | 1.30 | 1.72 | 8^h^ | 1.30 | 86.51 | 17.21 | 28.13 |
| Squacco Heron | *Ardeola ralloides* | predator | 2 | 18.5^l^ | 2.33 | -1.95 | 3.30 | 10^l^ | 0.13 | 86.40 | 15.32 | 20.20 |
| Tufted Titmouse | *Baeolophus bicolor* | predator | 4 | 78^s^ | 0.34 | -0.67 | 0.64 | 53^s^ | -0.45 | 15.84 | 8.71 | 15.73 |
| Cattle Egret | *Bubulcus ibis* | predator | 4 | 22.5^l^ | 1.82 | -1.28 | 2.05 | 10^l^ | 0.91 | 73.40 | 17.16 | 23.67 |
| Red-tailed Hawk | *Buteo jamaicensis* | raptor | 4 | 18^u^ | 1.87 | 0.85 | 0.47 | 18^u^ | 0.85 | 51.56 | 29.99 | 50.70 |
| Short-toed Snake Eagle | *Circaetus gallicus* | raptor | 2 | 15^m^ | 2.46 | 1.52 | 0.40 | 15^m^ | 1.52 | 62.51 | 42.81 | 66.03 |
| Rock Pigeon | *Columba livia* | pecking | 4 | 28^r^ | 1.52 | -0.22 | 1.14 | 19^r^ | 0.52 | 33.30 | 14.50 | 19.09 |
| Pied Crow | *Corvus albus* | pecking | 1 | 30^v^ | 1.24 | -0.34 | 1.14 | 8^v^ | 3.00 | 63.21 | 26.67 | 40.00 |
| American Crow | *Corvus brachyrhynchos* | pecking | 4 | 41^b^ | 0.88 | -0.93 | 1.22 | 37^b^ | -0.82 | 67.08 | 24.41 | 37.23 |
| Common Raven | *Corvus corax* | pecking | 4 | 34^v^ | 1.00 | -0.74 | 1.34 | 19^v^ | 0.07 | 82.85 | 29.15 | 47.67 |
| Carrion Crow | *Corvus corone* | pecking | 2 | 29^v^ | 1.18 | -0.34 | 1.21 | 11^v^ | 1.70 | 56.17 | 22.49 | 36.85 |
| Rook | *Corvus frugilegus* | pecking | 2 | 32^v^ | 1.13 | -0.48 | 1.18 | 13^v^ | 1.34 | 55.22 | 22.25 | 34.46 |
| Jackdaw | *Corvus monedula* | pecking | 2 | 40^v^ | 0.82 | -0.42 | 0.90 | 22^v^ | 0.28 | 36.39 | 17.59 | 29.46 |
| New Caledonian Crow | *Corvus moneduloides* | pecking | 1 | 40^v^ | 0.85 | -0.15 | 0.67 | 14^v^ | 1.50 | 55.90 | 34.50 | 55.44 |
| American Kestrel | *Falco sparverius* | raptor | 6 | 22^u^ | 1.77 | 0.82 | 0.47 | 22^u^ | 0.82 | 22.88 | 16.67 | 24.29 |
| Dark-eyed Junco | *Junco hyemalis* | pecking | 4 | 62^t^ | 0.48 | -0.82 | 0.73 | 36^t^ | -0.42 | 19.67 | 8.66 | 15.16 |
| Pink-eared Duck | *Malacorhynchus membranaceus* | tactile | 2 | 15^k^ | 2.43 | 0.05 | 1.66 | 15^k^ | 0.05 | 89.32 | 24.06 | 37.56 |
| Song Sparrow | *Melospiza melodia* | pecking | 2 | 49^t^ | 0.68 | -0.63 | 0.70 | 38^t^ | -0.41 | 20.55 | 9.69 | 15.75 |
| Black-crowned Night Heron | *Nycticorax nycticorax* | predator | 4 | 22^e^ | 1.86 | -1.15 | 2.09 | 13^e^ | 0.08 | 109.89 | 26.39 | 36.58 |
| Carolina Chickadee | *Parus carolinensis* | predator | 4 | 76^s^ | 0.36 | -0.61 | 0.63 | 51^s^ | -0.38 | 12.39 | 7.24 | 12.80 |
| Great Cormorant | *Phalacrocorax carbo* | predator | 2 | 24^q^ | 1.54 | -1.53 | 2.28 | 13^q^ | -0.12 | 94.74 | 20.19 | 30.94 |
| Lesser Flamingo | *Phoeniconaias minor* | tactile | 3 | 7^j^ | 6.36 | 1.76 | 3.50 | 7^j^ | 1.76 | 114.05 | 19.29 | 24.79 |
| Eastern Towhee | *Pipilo erythrophthalmus* | pecking | 2 | 67^t^ | 0.50 | -0.82 | 0.73 | 37^t^ | -0.34 | 24.89 | 12.39 | 18.90 |
| European Golden Plover | *Pluvialis apricaria* | pecking | 2 | 12^p^ | 3.27 | 1.45 | 1.36 | 12^p^ | 1.45 | 38.66 | 14.58 | 21.18 |
| Manx Shearwater | *Puffinus puffinus* | predator | 3 | 22^i^ | 1.57 | 0.24 | 0.96 | 16^i^ | 0.84 | 55.45 | 25.41 | 41.65 |
| Black Skimmer | *Rynchops niger* | tactile | 4 | 16^o^ | 2.31 | -1.05 | 2.71 | 16^o^ | -1.05 | 96.21 | 18.56 | 28.65 |
| Black Phoebe | *Sayornis nigricans* | predator | 1 | 40^c^ | 0.96 | -0.78 | 1.32 | 32^c^ | -0.53 | 23.62 | 9.45 | 13.58 |
| White-breasted Nuthatch | *Sitta carolinensis* | predator | 4 | 55^s^ | 0.54 | -1.34 | 1.23 | 45^s^ | -1.20 | 29.03 | 8.67 | 15.50 |
| American Tree Sparrow | *Spizella arborea* | pecking | 4 | 42^t^ | 0.78 | -0.55 | 0.89 | 38^t^ | -0.46 | 19.13 | 8.63 | 14.33 |
| Chipping Sparrow | *Spizella passerina* | pecking | 4 | 58^t^ | 0.54 | -0.76 | 0.71 | 38^t^ | -0.43 | 17.15 | 7.91 | 13.13 |
| Field Sparrow | *Spizella pusilla* | pecking | 2 | 57^t^ | 0.56 | -0.69 | 0.64 | 39^t^ | -0.39 | 17.04 | 8.33 | 13.66 |
| Ostrich | *Struthio camelus* | pecking | 2 | 20^n^ | 2.09 | 0.44 | 1.01 | 20^n^ | 0.44 | 118.96 | 53.15 | 72.02 |
| Eastern Meadowlark | *Sturnella magna* | pecking | 4 | 45^w^ | 0.74 | -0.86 | 1.16 | 32^w^ | -0.54 | 33.12 | 12.63 | 20.71 |
| European Starling | *Sturnus vulgaris* | pecking | 4 | 44^g^ | 0.75 | -0.90 | 1.31 | 22^g^ | -0.09 | 30.69 | 11.29 | 18.60 |
| Black-browed Albatross | *Thalassarche melanophrys* | predator | 1 | 22^f^ | 1.63 | -0.89 | 1.55 | 7^f^ | 2.93 | 148.33 | 37.33 | 58.73 |
| Mourning Dove | *Zenaida macroura* | pecking | 6 | 11^a^ | 3.77 | 2.10 | 1.04 | 9^a^ | 2.95 | 25.71 | 11.20 | 15.40 |
| White-throated Sparrow | *Zonotrichia albicollis* | pecking | 2 | 47^t^ | 0.70 | -0.39 | 0.68 | 39^t^ | -0.23 | 17.77 | 9.92 | 16.39 |

**S1 Table.** Data table for the 40 species used in analyses. Units for binocular field widths are in degrees. Unless specifically noted, values were calculated using converged binocular field widths. Units for the distance from focal point to peak tip, internodal distance, and skull width are in mm. Standardized parameters were divided by skull width to control for body size. Negative values of standardized blind gap indicate the ability to see the beak tip and positive values indicate the inability to see the beak tip. If no resting binocular state was reported in the literature, we calculated it as the extrapolated converged binocular – (0.33 * (extrapolated converged binocular – diverged binocular)) which is the proportion for starlings in Martin 1986.

REFERENCES FOR SUPPLEMENTARY MATERIAL

a. Blackwell BF, Fernández-Juricic E, Seamans TW, Dolan T. Avian visual system configuration and behavioural response to object approach. Anim Behav. 2009; 77: 673–684.

b. Fernández-Juricic E, O’Rourke C, Pitlik T. Visual coverage and scanning behavior in two corvid species: American crow and Western scrub jay. J Comp Physiol A. 2010; 196: 879–888.

c. Gall MD, Fernández-Juricic E. Visual fields, eye movements, and scanning behavior of a sit-and-wait predator, the black phoebe (*Sayornis nigricans*). J Comp Physiol A. 2010; 196: 15–22.

d. Guillemain M, Martin GR, Fritz H. Feeding methods, visual fields and vigilance in dabbling ducks (Anatidae). Funct Ecol. 2002; 16: 522–529.

Jetz W, Thomas GH, Joy JB, et al. The global diversity of birds in space and time. Nature. 2012; 491: 444–448.

e. Katzir G, Martin GR. Visual fields in the black-crowned night heron Nycticorax nycticorax: nocturnality does not result in owl-like features. Ibis. 1998; 140: 157–162.

f. Martin GR. Eye structure and amphibious foraging in albatrosses. Proc R Soc London B. 1998; 265: 665–671.

g. Martin GR. The eye of a passeriform bird, the European starling (*Sturnus vulgaris*): eye movement amplitude, visual fields and schematic optics. J Comp Physiol A. 1986; 159: 545–557.

h. Martin GR. Total panoramic vision in the mallard duck, *Anas platyrhynchos*. Vision Res. 1986; 26: 1303–1305.

i. Martin GR, Brooke M de L. The eye of a procellariiform seabird, the manx shearwater, *Puffinus puffinus*: visual fields and optical structure. Brain Behav Evol. 1991; 37: 65–78.

j. Martin GR, Jarrett N, Tovey P, White CR. Visual fields in Flamingos: Chick-feeding versus filter-feeding. Naturwissenschaften. 2005; 92: 351–354.

k. Martin GR, Jarrett N, Williams MJ. Visual fields in Blue Ducks *Hymenolaimus malacorhynchos* and Pink-eared Ducks *Malacorhynchus membranaceus*: visual and tactile foraging. Ibis 2007; 149: 112–120.

l. Martin GR, Katzir G. Visual fields and eye movements in herons (Ardeidae). Brain Behav Evol. 1994; 44: 74–85.

m. Martin GR, Katzir G. Visual fields in short-toed eagles, *Circaetus gallicus* (Accipitridae), and the function of binocularity in birds. Brain Behav Evol. 1999; 53: 55–66.

n. Martin GR, Katzir G. Visual fields in ostriches. Nature. 1994; 374: 19–20.

o. Martin GR, McNeil R, Rojas LM. Vision and the foraging technique of skimmers (Rynchopidae). Ibis 2007; 149: 750–757.

p. Martin GR, Piersma T. Vision and touch in relation to foraging and predator detection: insightful contrasts between a plover and a sandpiper. Proc R Soc London B. 2009; 276: 437–445.

q. Martin GR, White CR, Butler PJ. Vision and the foraging technique of Great Cormorants *Phalacrocorax carbo*: pursuit or close-quarter foraging? Ibis 2008; 150: 485–494.

r. Martin GR, Young SR. The retinal binocular field of the pigeon (*Columba livia*: English racing homer). Vision Res 1983; 23: 911–915.

s. Moore BA, Doppler M, Young JE, Fernández-Juricic E. Interspecific differences in the visual system and scanning behavior of three forest passerines that form heterospecific flocks. J Comp Physiol A 2013; 199: 263–277.

t. Moore BA, Pita D, Tyrrell LP, Fernandez-Juricic E. Vision in avian emberizid foragers: maximizing both binocular vision and fronto-lateral visual acuity. J Exp Biol. 2015; 218: 1347–1358.

u. O’Rourke CT, Hall MI, Pitlik T, Fernández-Juricic E. Hawk eyes I: diurnal raptors differ in visual fields and degree of eye movement. PLoS One. 2010; 5: e12802.

Rubolini D, Liker A, Garamszegi LZ, et al. Using the BirdTree.org website to obtain robust phylogenies for avian comparative studies: a primer. Curr Zool 2015; 61: 959–965.

Sukumaran J, Holder MT. DendroPy: A Python library for phylogenetic computing. Bioinformatics. 2010; 26: 1569–1571.

v. Troscianko J, von Bayern AMP, Chappell J, et al. Extreme binocular vision and a straight bill facilitate tool use in New Caledonian crows. Nat Commun. 2012; 3: 1110.

w. Tyrrell LP, Moore BA, Loftis C, Fernández-Juricic E. Looking above the prairie: localized and upward acute vision in a native grassland bird. Sci Rep. 2013; 3: 3231.

SPECIMEN INFORMATION

No live animals were used in this study. All visual field data was gathered from previously published literature. All morphological measurements were obtained from specimens that are accessible in the Bird Specimen Collection permanent repository at the Field Museum of Natural History in Chicago, IL, USA. Specimen numbers can be found below.

| **Common name** | **Species name** | **Specimen ID** | **Eye to beak tip (mm)** | **Beak length (mm)** | | **internodal distance (mm)** | **Skull width (mm)** | **Sex** |
| --- | --- | --- | --- | --- | --- | --- | --- | --- |
| Cooper's Hawk | *Accipiter cooperii* | 496838 | 37.82 | 17.42 | | 24.78 | 37.7 | female |
| Cooper's Hawk | *Accipiter cooperii* | 498646 | 31.39 | 16.28 | | 24 | 31.63 | male |
| Cooper's Hawk | *Accipiter cooperii* | 492437 | 31.22 | 16.05 | | 22.81 | 29.46 | male |
| Cooper's Hawk | *Accipiter cooperii* | 495206 | 35.05 | 17.56 | | 23.91 | 33.38 | female |
| Northern Shoveler | *Anas clypeata* | 339047 | 98.09 | 57.87 | | 13.31 | 21.83 | male |
| Northern Shoveler | *Anas clypeata* | 436228 | 88.91 | 53.68 | | 12.79 | 20.49 | female |
| Northern Shoveler | *Anas clypeata* | 351036 | 85.35 | 52.16 | | 14.43 | 20.69 | female |
| Northern Shoveler | *Anas clypeata* | 496821 | 96.21 | 58.65 | | 13.9 | 21.98 | male |
| Eurasian Wigeon | *Anas penelope* | 104076 | 65.05 | 31.55 | | 13.93 | 23.65 | female |
| Eurasian Wigeon | *Anas penelope* | 105593 | 63.53 | 31.62 | | 13.24 | 24.68 |  |
| Mallard | *Anas platyrhynchos* | 498630 | 91.44 | 50.1 | | 17.06 | 28.18 | male |
| Mallard | *Anas platyrhynchos* | 496454 | 93.24 | 52.59 | | 17.26 | 28.99 | male |
| Mallard | *Anas platyrhynchos* | 488415 | 77.15 | 44.12 | | 15.91 | 26.64 | female |
| Mallard | *Anas platyrhynchos* | 495170 | 84.19 | 47.15 | | 18.62 | 28.7 | female |
| Squacco Heron | *Ardeola ralloides* | 313700 | 86.72 | 67.42 | | 15.99 | 20.27 | male |
| Squacco Heron | *Ardeola ralloides* | 313699 | 86.08 | 65.97 | | 14.65 | 20.13 |  |
| Tufted Titmouse | *Baeolophus bicolor* | 336414 | 14.94 | 8.61 | | 8.23 | 15.29 | female |
| Tufted Titmouse | *Baeolophus bicolor* | 336413 | 15.33 | 10.09 | | 8.82 | 15.55 | male |
| Tufted Titmouse | *Baeolophus bicolor* | 395353 | 18.43 | 12.16 | | 9 | 16.22 | male |
| Tufted Titmouse | *Baeolophus bicolor* | 377118 | 14.65 | 9.28 | | 8.77 | 15.86 | female |
| Cattle Egret | *Bubulcus ibis* | 339299 | 72.46 | 48.25 | | 16.52 | 24.05 | male |
| Cattle Egret | *Bubulcus ibis* | 339304 | 76.61 | 53.27 | | 17.37 | 24.13 | male |
| Cattle Egret | *Bubulcus ibis* | 339297 | 71.46 | 47.31 | | 18.1 | 22.88 | female |
| Cattle Egret | *Bubulcus ibis* | 375719 | 73.06 | 44.96 | | 16.63 | 23.63 | female |
| Red-tailed Hawk | *Buteo jamaicensis* | 495229 | 48.75 | 20.75 | | 29.72 | 48.53 | male |
| Red-tailed Hawk | *Buteo jamaicensis* | 495235 | 55.48 | 25.96 | | 29.99 | 53.08 | female |
| Red-tailed Hawk | *Buteo jamaicensis* | 496844 | 51.77 | 23.33 | | 29.44 | 50.24 | female |
| Red-tailed Hawk | *Buteo jamaicensis* | 498262 | 50.25 | 25.14 | | 30.81 | 50.93 | male |
| Short-toed Snake Eagle | *Circaetus gallicus* | 106719 | 60.57 | 25.32 | | 43.79 | 66.26 |  |
| Short-toed Snake Eagle | *Circaetus gallicus* | 106722 | 64.45 | 27.2 | | 41.83 | 65.79 |  |
| Rock Pigeon | *Columba livia* | 500181 | 32.16 | 22.12 | | 14.29 | 19.32 | female |
| Rock Pigeon | *Columba livia* | 496946 | 32.45 | 21.41 | | 13.8 | 19.53 | female |
| Rock Pigeon | *Columba livia* | 498226 | 32.4 | 20.52 | | 15.23 | 16.6 | male |
| Rock Pigeon | *Columba livia* | 495102 | 36.19 | 23.29 | | 14.66 | 20.91 | male |
| Pied Crow | *Corvus albus* | 335648 | 63.21 | 45.45 | | 26.67 | 40 | female |
| American Crow | *Corvus brachyrhynchos* | 441516 | 70.04 | 47.52 | | 24.12 | 37.35 | male |
| American Crow | *Corvus brachyrhynchos* | 437427 | 76.81 | 51.42 | | 24.48 | 38.83 | male |
| American Crow | *Corvus brachyrhynchos* | 428922 | 60.9 | 40.78 | | 24.24 | 35.77 | female |
| American Crow | *Corvus brachyrhynchos* | 428924 | 60.57 | 42.1 | | 24.79 | 36.95 | female |
| Common Raven | *Corvus corax* | 390650 | 80.75 | 62.72 | | 29.89 | 45.6 | female |
| Common Raven | *Corvus corax* | 365062 | 83.59 | 65.68 | | 29 | 48.11 | male |
| Common Raven | *Corvus corax* | 465345 | 81.23 | 63.62 | | 27.9 | 48.1 | female |
| Common Raven | *Corvus corax* | 488640 | 85.84 | 64.09 | | 29.82 | 48.87 | male |
| Carrion Crow | *Corvus corone* | 335644 | 56.56 | 45.91 | | 22.6 | 37.92 | unknown |
| Carrion Crow | *Corvus corone* | 335643 | 55.77 | 43.02 | | 22.38 | 35.78 | unknown |
| Rook | *Corvus frugilegus* | 335631 | 54.37 | 40.54 | | 21.45 | 34.05 | unknown |
| Rook | *Corvus frugilegus* | 335630 | 56.07 | 40.45 | | 23.05 | 34.87 | unknown |
| Jackdaw | *Corvus monedula* | 335626 | 36.96 | 27.81 | | 18.19 | 29.46 | female |
| Jackdaw | *Corvus monedula* | 335625 | 35.82 | 25.49 | | 16.99 | 29.46 | male |
| New Caledonian Crow | *Corvus monoduloides* | skin | 55.9 | 37.4 | | 34.5 | 55.44 | unknown |
| American Kestrel | *Falco sparverius* | 428820 | 22.74 | 12.72 | | 14.97 | 23.06 | male |
| American Kestrel | *Falco sparverius* | 475306 | 24 | 12.67 | | 16.36 | 24.9 | female |
| American Kestrel | *Falco sparverius* | 379056 | 21.14 | 8.52 | | 18.21 | 24.51 | male |
| American Kestrel | *Falco sparverius* | 468640 | 23.85 | 12.35 | | 16.67 | 23.5 | female |
| American Kestrel | *Falco sparverius* | 436589 | 23.42 | 10.29 | | 18.1 | 26.84 | female |
| American Kestrel | *Falco sparverius* | 435677 | 22.13 | 11.63 | | 15.71 | 22.92 | male |
| Dark-eyed Junco | *Junco hyemalis* | 467381 | 20.44 | 11.09 | | 8.59 | 15.35 | male |
| Dark-eyed Junco | *Junco hyemalis* | 467378 | 19.84 | 10.87 | | 9.08 | 14.61 | male |
| Dark-eyed Junco | *Junco hyemalis* | 467379 | 19.11 | 10.93 | | 8.45 | 15.25 | female |
| Dark-eyed Junco | *Junco hyemalis* | 467380 | 19.3 | 11.23 | | 8.51 | 15.42 | female |
| Pink-eared Duck | *Malacorhynchos membranaceus* | 7581 | 96.16 | 63.28 | | 20.34 | 38.4 | unknown |
| Pink-eared Duck | *Malacorhynchos membranaceus* | skin | 82.47 | 61.24 | | 27.77 | 36.8 | unknown |
| Song Sparrow | *Melospiza melodia* | 384046 | 19.62 | 9.95 | | 9.6 | 15.62 | male |
| Song Sparrow | *Melospiza melodia* | 386037 | 21.47 | 11.96 | | 9.78 | 15.87 | female |
| Black-crowned Night Heron | *Nycticorax nycticorax* | 341924 | 113.67 | 80.98 | | 23.76 | 36.06 | male |
| Black-crowned Night Heron | *Nycticorax nycticorax* | 483845 | 115.6 | 81.92 | | 25.68 | 36.14 | male |
| Black-crowned Night Heron | *Nycticorax nycticorax* | 378445 | 101.98 | 69.32 | | 28.66 | 36.53 | female |
| Black-crowned Night Heron | *Nycticorax nycticorax* | 342340 | 108.32 | 73.69 | | 27.44 | 37.59 | female |
| Great Cormorant | *Phalacrocorax carbo* | 339390 | 105.95 | 78.55 | | 22.5 | 33.1 | female |
| Great Cormorant | *Phalacrocorax carbo* | 368739 | 83.52 | 62.57 | | 17.88 | 28.78 | female |
| Lesser Flamingo | *Phoeniconaias minor* | 339182 | 113.05 | 86.26 | | 18.51 | 22.28 | male |
| Lesser Flamingo | *Phoeniconaias minor* | 339185 | 118.04 | 90.38 | | 19.54 | 22.83 | male |
| Lesser Flamingo | *Phoeniconaias minor* | 339184 | 111.07 | 84 | | 19.81 | 29.27 | female |
| Eastern Towhee | *Pipilo erythrophthalmus* | 435649 | 24.5 | 13.84 | | 12.88 | 18.91 | male |
| Eastern Towhee | *Pipilo erythrophthalmus* | 318088 | 25.28 | 13.81 | | 11.9 | 18.88 | female |
| European Golden Plover | *Pluvialis apricaria* | 376148 | 40.94 | 31.82 | | 14.84 | 21.29 | male |
| European Golden Plover | *Pluvialis apricaria* | 338392 | 36.37 | 25.79 | | 14.32 | 21.07 |  |
| Carolina Chickadee | *Poecile carolinensis* | 336404 | 11.12 | 6.58 | | 7.67 | 12.87 | male |
| Carolina Chickadee | *Poecile carolinensis* | 432602 | 13.61 | 9.24 | | 6.94 | 13.44 | male |
| Carolina Chickadee | *Poecile carolinensis* | 440346 | 11.64 | 7.43 | | 6.9 | 11.79 |  |
| Carolina Chickadee | *Poecile carolinensis* | 428665 | 13.2 | 9.01 | | 7.43 | 13.11 |  |
| Manx Shearwater | *Puffinus puffinus* | skin | 55.13 | 40.22 | | 24.43 | 42.15 | unknown |
| Manx Shearwater | *Puffinus puffinus* | skin | 55.76 | 40.12 | | 26.39 | 42.7 | unknown |
| Manx Shearwater | *Puffinus puffinus* | skin | 55.12 | 39.91 | | 23.88 | 40.1 | unknown |
| Black Skimmer | *Rynchops niger* | 376311 | 102.97 | 84.72 | | 17.17 | 28.12 | female |
| Black Skimmer | *Rynchops niger* | 376314 | 77.76 | 61.65 | | 16.77 | 25.5 | female |
| Black Skimmer | *Rynchops niger* | 398882 | 113.68 | 91.82 | | 20.01 | 30.79 | male |
| Black Skimmer | *Rynchops niger* | 338005 | 90.41 | 72.13 | | 20.29 | 30.17 | male |
| Black Phoebe | *Sayornis nigricans* | 317866 | 23.62 | 17.94 | | 9.45 | 13.58 |  |
| White-breasted Nuthatch | *Sitta carolinensis* | 437356 | 29.63 | 18.89 | | 8.95 | 15.41 | male |
| White-breasted Nuthatch | *Sitta carolinensis* | 435445 | 29.3 | 19.19 | | 9.14 | 15.81 | male |
| White-breasted Nuthatch | *Sitta carolinensis* | 440138 | 29.21 | 19.68 | | 8.31 | 15.57 | female |
| White-breasted Nuthatch | *Sitta carolinensis* | 443491 | 27.96 | 18.29 | | 8.27 | 15.21 | female |
| American Tree Sparrow | *Spizella arborea* | 436957 | 19.43 | 10.46 | | 8.18 | 14.44 | male |
| American Tree Sparrow | *Spizella arborea* | 436953 | 19.25 | 19.25 | | 8.99 | 14.65 | male |
| American Tree Sparrow | *Spizella arborea* | 436954 | 18.55 | 10.77 | | 8.61 | 14.12 | female |
| American Tree Sparrow | *Spizella arborea* | 436955 | 19.27 | 10.59 | | 8.72 | 14.12 | female |
| Chipping Sparrow | *Spizella passerina* | 340065 | 18.06 | 9.88 | | 7.76 | 13.33 | male |
| Chipping Sparrow | *Spizella passerina* | 361013 | 17.16 | 9.34 | | 7.5 | 12.69 | male |
| Chipping Sparrow | *Spizella passerina* | 383050 | 16.11 | 8.67 | | 8.53 | 13.38 | female |
| Chipping Sparrow | *Spizella passerina* | 340066 | 17.25 | 9.36 | | 7.85 | 13.11 | female |
| Field Sparrow | *Spizella pusilla* | 333082 | 17.7 | 9.31 | | 8.32 | 13.98 | male |
| Field Sparrow | *Spizella pusilla* | 333080 | 16.37 | 8.3 | | 8.33 | 13.33 | female |
| Eastern Meadowlark | *Sternella magna* | 88425 | 33.07 | 23.74 | | 13.48 | 21.8 | female |
| Eastern Meadowlark | *Sternella magna* | 494969 | 33.49 | 24.46 | | 12.73 | 20.75 | male |
| Eastern Meadowlark | *Sternella magna* | 348145 | 31.2 | 22.08 | | 11.98 | 19.76 | male |
| Eastern Meadowlark | *Sternella magna* | 493373 | 34.7 | 25.47 | | 12.31 | 20.52 | female |
| European Starling | *Sturnus vulgaris* | 488405 | 30.12 | 22.64 | | 10.41 | 16.91 | female |
| European Starling | *Sturnus vulgaris* | 466186 | 36.1 | 27.33 | | 12.95 | 19.09 | male |
| European Starling | *Sturnus vulgaris* | 470240 | 21.58 | 21.37 | | 11.46 | 18.66 | female |
| European Starling | *Sturnus vulgaris* | 477254 | 34.95 | 26.42 | | 10.34 | 19.75 | male |
| Ostrich | *Sturthio camelus* | 378755 | 114.92 | 72.72 | | 43.3 | 72.02 | unknown |
| Ostrich | *Sturthio camelus* | full mount | 123 |  | | 63 |  | unknown |
| Black-browed Albatross | *Thalassarche melanophris* | 339602 | 148.33 | 91.27 | | 37.33 | 58.73 |  |
| Mourning Dove | *Zenaida macroura* | 337943 | 27.77 | 16.88 | | 11.99 | 15.56 | male |
| Mourning Dove | *Zenaida macroura* | 337940 | 24.6 | 15.03 | | 11.98 | 15.87 | female |
| Mourning Dove | *Zenaida macroura* | 337941 | 26.43 | 16.55 | | 11.37 | 15.39 | female |
| Mourning Dove | *Zenaida macroura* | 390256 | 28.14 | 16.9 | | 12.45 | 16.38 | male |
| Mourning Dove | *Zenaida macroura* | 427673 | 27.18 | 16.08 | | 11.36 | 15.12 | female |
| Mourning Dove | *Zenaida macroura* | 390438 | 20.16 | 15.03 | | 8.02 | 14.1 | male |
| White-throated Sparrow | *Zonotrichia albicollis* | 317565 | 18.08 | 10.7 | | 9.92 | 16.17 | male |
| White-throated Sparrow | *Zonotrichia albicollis* | 317574 | 17.46 | 11.74 | | 9.91 | 16.6 | female |
